# Specificity, Safety, Efficacy of EGFRvIII-Retargeted Oncolytic HSV for Xenotransplanted Human Glioblastoma

**DOI:** 10.3390/v13091677

**Published:** 2021-08-24

**Authors:** Irene Appolloni, Francesco Alessandrini, Laura Menotti, Elisa Avitabile, Daniela Marubbi, Noemi Piga, Davide Ceresa, Francesca Piaggio, Gabriella Campadelli-Fiume, Paolo Malatesta

**Affiliations:** 1 Department of Experimental Medicine (DIMES), University of Genova, 16132 Genova, Italy; irene.appolloni@unige.it (I.A.); daniela.marubbi@unige.it (D.M.); noemipiganicole@gmail.com (N.P.); francesca.piaggio@hsanmartino.it (F.P.); 2 IRCCS Ospedale Policlinico San Martino, 16132 Genova, Italy; francescoalessandrini89@gmail.com (F.A.); davide.ceresa91@gmail.com (D.C.); 3 Department of Pharmacy and Biotechnology, University of Bologna, 40126 Bologna, Italy; laura.menotti@unibo.it (L.M.); elisa.avitabile@unibo.it (E.A.); 4 Department of Experimental, Diagnostic and Specialty Medicine, University of Bologna, 40126 Bologna, Italy; gabriella.campadelli@unibo.it

**Keywords:** oncolytic virotherapy, glioblastoma stem cells, glioblastoma initiating cells, EGFRvIII, oncolytic HSV

## Abstract

Glioblastoma is a lethal primary brain tumor lacking effective therapy. The secluded onset site, combined with the infiltrative properties of this tumor, require novel targeted therapies. In this scenario, the use of oncolytic viruses retargeted to glioblastoma cells and able to spread across the tumor cells represent an intriguing treatment strategy. Here, we tested the specificity, safety and efficacy of R-613, the first oncolytic HSV fully retargeted to EGFRvIII, a variant of the epidermal growth factor receptor carrying a mutation typically found in glioblastoma. An early treatment with R-613 on orthotopically transplanted EGFRvIII-expressing human glioblastoma significantly increased the median survival time of mice. In this setting, the growth of human glioblastoma xenotransplants was monitored by a secreted luciferase reporter and showed that R-613 is able to substantially delay the development of the tumor masses. When administered as late treatment to a well-established glioblastomas, R-613 appeared to be less effective. Notably the uninfected tumor cells derived from the explanted tumor masses were still susceptible to R-613 infection ex vivo, thus suggesting that multiple treatments could enhance R-613 therapeutic efficacy, making R-613 a promising oncolytic HSV candidate for glioblastoma treatment.

## 1. Introduction

Gliomas are the most common primary malignant brain tumors. Glioblastoma represents the most aggressive form, with poor prognosis, and leads to patient death with a median survival time of about 15 months from diagnosis [1]. Glioblastoma poses huge challenges to modern medicine. Its highly infiltrative behavior hinders a complete surgical resection. It easily develops resistance to radio- and chemotherapy and undermines standard therapies. In recent decades, many innovative strategies have been proposed to improve patient conditions [2], including neurologic function, overall survival, psychological health and quality of life. Among many different approaches, oncolytic virotherapy appears to be a very promising tool [3,4]. First of all, oncolytic viruses (OVs) are designed to selectively target and kill tumor cells. Importantly, OVs replicate specifically in cancer cells, generate and release viral progeny that can infect neighboring malignant cells, amplifying the therapeutic effect. Furthermore, OVs can be genetically modified (armed) to deliver pro-drugs activating genes, anti-angiogenic or immune stimulating genes [5,6]. Finally, the virus itself acts as an immunological adjuvant triggering immune response [7,8], potentially favoring an agnostic vaccination against tumor associated antigens (TAA) released following the lysis of tumor cells [9,10]. This immunotherapeutic effect is of paramount importance especially in immunosuppressive tumors as glioblastomas, where cancer cells progressively become stealth to the immune system and a pro-tumorigenic immune infiltrate prevails [11,12]. The efficacy of anti-tumor immune stimulation by OVs has been extensively reported in glioma treatment [13,14]. In addition, OVs are able to diffuse through the brain parenchyma, allowing them to also reach glioma cells far from the tumor mass [15]. This feature is crucial to counteract glioblastoma, a tumor with a markedly migratory phenotype.

Many different OVs have been tested to treat glioblastoma, including adenovirus, herpes simplex virus (HSV), vaccinia virus [16,17]. Some of them have, in fact, entered phase II or III clinical trials [14,18,19,20]. Among all OVs, oncolytic HSVs (oHSVs) appear to be the most promising. HSV advantages encompass its high cytolytic effect, its large and stable genome that can be easily engineered, its ability to grow at high titres and the availability of specific anti-viral drugs to counter any possible adverse reactions [6,21]. In many instances, oHSV selectivity for cancer cells has been achieved generating attenuated viruses, carrying deletion of specific genes necessary for virus replication in non-dividing cells and dispensable for viral replication in proliferating tumor cells. In recent years, such genetically modified attenuated oHSVs were assayed in a number of clinical trials and are well tolerated by patients [14,17,18,19,22,23]. The oHSV Talimogene laherparepvec (T-Vec) was the first OV to be approved by the FDA and EMA for melanoma treatment [24].

The attenuating modifications described above improve the safety of the treatment but, at the same time, reduce the viral replicative and lytic ability, thus limiting the therapeutic potential [6,25]. *dlsptk*, the first genetically engineered oHSV tested for glioblastoma treatment carried the deletion of the thymidine kinase gene, improved survival of treated mice and boosted research in the field of oncolytic virotherapy [26]. Many more attenuated oHSV were developed [21]. Most of them were engineered with deletion in both copies of the γ_1_34.5 neurovirulence gene and possibly additional deletions [6]: oHSV G207, lacking the two copies of γ_1_34.5 and UL39 genes, showed an increased glioblastoma selectivity, and oHSV G47Δ, with an additional deletion in US12, had an increased viral replication in glioblastoma cells [27,28]. Other strategies to rescue viral replication in attenuated Δγ_1_34.5 oHSVs included the re-insertion of the γ_1_34.5 gene under the nestin promoter (oHSV rQNestin34.5v.2) to prevent replication in differentiated neural cells and limit it to glioblastoma cells [29]. In other recombinant oHSVs, to complement γ_1_34.5 deletion and maintain glioblastoma specificity, heterologous genes as the human GADD34 gene (for oHSV NG34) or the IRS1 gene from cytomegalovirus (for oHSV C134) were engineered into the viral genome under the control of the nestin promoter [30,31].

Despite the variety of the strategies employed, attenuated safe and glioma-specific oHSVs showed a reduced replication and lytic potential with respect to wild-type viruses, implying limitations in their therapeutic efficacy. A more recent strategy devised to overcome this limitation is based on fully virulent viruses that are totally detargeted from their natural entry receptors and specifically retargeted to tumor cells [18,32,33]. Detargeting and retargeting is usually obtained by mutations on envelope glycoproteins essential for virus entry, that consist in deletions and/or insertion of heterologous ligands (e.g., single chain antibodies, scFv) against receptors specifically expressed by tumor cells [21].

EGFR and HER2 receptors are expressed at high levels in human glioblastomas and other solid tumors [34,35,36] and have been chosen as targets for a number of oHSVs engineered with specific scFvs (for a review, see [21]). In particular, KNE, an oHSV retargeted to human EGFR, significantly increased the survival of mice orthotopically injected with human glioblastoma cells and was able to cure about 70% of treated animals [37]. We recently demonstrated the preclinical efficacy of a fully virulent oHSV, R-LM113, retargeted to the human HER2 receptor [15,38]). The treatment with R-LM113 was able to double the survival time of animals bearing HER2-positive glioblastomas. The therapeutic efficacy was further improved by treating mice with oHSV R-115, a derivative recombinant armed with murine interleukin 12 (mIL12); glioblastomas were eradicated in 30% of the animals and an immunological memory to the tumor was induced and conferred immunization against rechallenge [39].

Although HER2 and EGFR receptors are expressed in human gliomas and correlate with malignancy [34,35,40,41], they do not represent distinctive glioma markers. In order to increase glioma selectivity, some authors exploit the differences in miRNA expression in glioma versus healthy neural cells. As an example, miR-124 is expressed in healthy neural cells and absent in gliomas [42,43]. KGE-4:T124, a KNE-derivative that contains miR-124 recognition site in the 3′-UTR of one copy of the viral ICP4 gene, was proven safe when injected in mouse brains, and showed high levels of viral replication in glioblastoma cells [44]. However, a concern about the use of this oHSV remains, due to the lack of miR-124 expression in neural stem cells that are positive for EGFR, which could represent a potential off-target for this EGFR-retargeted oHSV [45,46].

In this scenario, a very interesting target is represented by EGFRvIII, a constitutively active variant of the epithelial growth factor receptor that is specifically and widely expressed in glioblastomas [35,47]. A number of therapies directed against EGFRvIII are currently in pre-clinical or clinical phase of investigation. They include, but are not limited to, vaccines [48,49], antibodies [50,51] and chimeric antigen receptor (CAR) T cells [52,53]. We recently developed and described a new fully virulent recombinant oHSV retargeted to EGFRvIII, R-613 [33], and here we tested its safety and therapeutic efficacy on patient-derived human glioblastoma initiating cells (hGIC), both in vitro and in a mouse preclinical model.

## 2. Materials and Methods

### 2.1. Cell Cultures and R-613 Infection

Engineering of murine glioblastoma cells expressing EGFRvIII (HGG-E, Supplementary Table S1) was previously described [54]. Tumor dissociation and maintenance was performed as previously described [11]. Dissociated cells were cultured on Matrigel coated flasks (1:200; BD Biosciences) in DMEM/F12 (Life Technologies, Carlsbad, CA, USA), supplemented with B27 supplement (Life Technologies, Carlsbad, CA, USA), 2 mM L-glutamine (Life Technologies, Carlsbad, CA, USA) penicillin/streptomycin (Life Technologies, Carlsbad, CA, USA), 10 ng/mL basic FGF (Peprotech, Cranbury, NJ, USA) and 20 ng/mL EGF (Peprotech, Cranbury, NJ, USA).

Human glioblastoma cells derived by glioblastoma patient (kindly provided by Dr. Rossella Galli, IRCCS San Raffaele Scientific Institute, Supplementary Table S1) were maintained on Matrigel (1:200; BD Biosciences) coated flasks in Neurocult NS-A basal media supplemented with Neurocult proliferation supplement (Stem Cell Technologies, Cologne, Germany), 10 ng/mL recombinant human basic FGF (Peprotech, Cranbury, NJ, USA), 20 ng/mL recombinant human EGF (Peprotech, Cranbury, NJ, USA), heparin 2 µg/mL (Sigma-Aldrich, St. Louis, MO, USA).

Human glioblastoma cells derived by glioblastoma patient (kindly provided by Dr. Antonio Daga, Ospedale Policlinico San Martino, Supplementary Table S1) were maintained on Matrigel (1:200; BD Biosciences) coated flasks in 50% Neurobasal media, 50% DMEM/F12 (Life Technologies, Carlsbad, CA, USA), supplemented with B27 supplement (Life Technologies, Carlsbad, CA, USA), 2 mM L-glutamine (Life Technologies, Carlsbad, CA, USA) penicillin/streptomycin (Life Technologies, Carlsbad, CA, USA), 10 ng/mL basic FGF (Peprotech, Cranbury, NJ, USA) and 20 ng/mL EGF (Peprotech, Cranbury, NJ, USA), heparin 2 µg/mL (Sigma-Aldrich, St. Louis, MO, USA).

oHSV R-613 viral stocks and purified extracellular virions (Supplementary Table S2) were produced in U251-EGFRvIII cells and harvested as described [55,56]. Purified extracellular virions were used for in vivo treatments in animal brains, viral stocks for in vitro infections.

Cell infection was performed by inoculating glioblastoma cell cultures with oHSV R-613 [33] at different MOI, calculated on the basis of the viral titer determined in U251-EGFRvIII. The percentage of infected cells was analyzed by flow cytometry (CyAn ADP, Beckman Coulter, Indianapolis, IN, USA) reading EGFP fluorescence intensity. Images for the analysis of viral cell-to-cell spread in vitro were obtained by capturing multiple images over time under an inverted fluorescence microscope (EVOS FL Cell Imaging System, ThermoFisher Scientific, Waltham, MA, USA).

### 2.2. Animal Procedures

All the animal procedures were approved by the Ethical Committee for Animal Experimentation (CSEA) of the IRCCS Ospedale Policlinico San Martino, Genova, and by the Italian Ministry of Health (N° 859/2016-PR). Animals were handled following Italian current regulations about animal use for scientific purposes (D.lvo 27 January 1992, n. 116). In all the experiments the NOD.CB17-Prkdcscid/NCrHsd strain (Envigo), hereinafter referred to as NOD/SCID, was used.

Cells injections in the brain parenchyma of adult mice were performed as described in a previous study [57]. Briefly, up to 5 μL, containing 1–2 × 10^5^ L0306 human glioblastoma cells, suspended in Neurocult medium, were intracranially injected in adult mouse brains. Injections were performed by using a stereotaxic apparatus and a Hamilton syringe at the following coordinates from Bregma: 1.0 mm anterior, 1.0 mm left and 2.5 mm below the skull surface. In the late treatment condition, a minimum of 10^9^ PFU of oHSV R-613 purified extracellular virions, diluted in DMEM + 10% FBS medium, were injected at the same coordinates 42 days after cell transplant. For the double treatment, the second injection was scheduled at 64 days after cell transplant. DMEM + 10% FBS medium alone was injected into control mice.

To measure tumor growth in vivo, 8 μL of blood were drawn from the tail tip and harvested in 2 μL of 0.5 M EDTA. Gluc quantification was performed using the Dual-Luciferase Reporter Assay System kit (Promega) and the GloMax20/20 luminometer (Promega). Final measurements were obtained using a R script (available on request) to calculate the level of Gluc as described in [58].

Dissected brains were analyzed under a fluorescence stereo-microscope (Leica MZ10F) to visualize DsRed tumor masses and EGFP-positive R-613 infected areas.

### 2.3. Western Blot

Cells were harvested in lysis buffer containing 50 mM HEPES (pH 7.5), 5 mM EDTA, 150 mM NaCl, 1% Triton X-100 detergent, 1 mM sodium orthovanadate, 5 mM sodium fluoride and protease inhibitors (Complete, Roche Applied Science). Protein levels were normalized to α-tubulin protein level. Primary antibodies: EP38Y anti-EGFR rabbit monoclonal antibody (1:1000, AbCam, Cambridge, UK), anti-α-tubulin mouse monoclonal antibody (1:5000, Sigma, St. Louis, MO, USA); secondary antibodies: HRP-conjugated anti-mouse and anti-rabbit antibodies (1:5000, Sigma, St. Louis, MO, USA).

### 2.4. Statistical Analysis

Mice were randomly allocated to the different experimental groups by assigning them a unique ID and using the Microsoft Excel pseudorandom generator to sort them. Animals that did not survive intracranial injection procedure and died within 3 days after virus injection were excluded from the experiment. Survival curves were compared using log Rank Test. All statistical analyses were performed in R environment (R Foundation for Statistical Computing, Vienna, Austria).

## 3. Results

### 3.1. EGFRvIII Retargeted oHSV Is Highly Specific for EGFRvIII Expressing Glioblastoma Cells

We recently designed and engineered an oHSV, named R-613, completely detargeted from its natural receptors, and retargeted to EGFRvIII, a mutated form of the epidermal growth factor, typical of human glioblastoma [33]. Full retargeting was achieved by the insertion of a single chain antibody (scFv) in the viral envelope glycoprotein D (gD) and deletion in gD which abrogate interaction with nectin and HVEM. In addition, R-613 expresses EGFP, for the detection and quantification of infection. Previously, we showed that R-613 is able to infect U251 glioblastoma cells overexpressing EGFRvIII, while it does not infect the parental U251 cell line [33].

**Figure 1:**
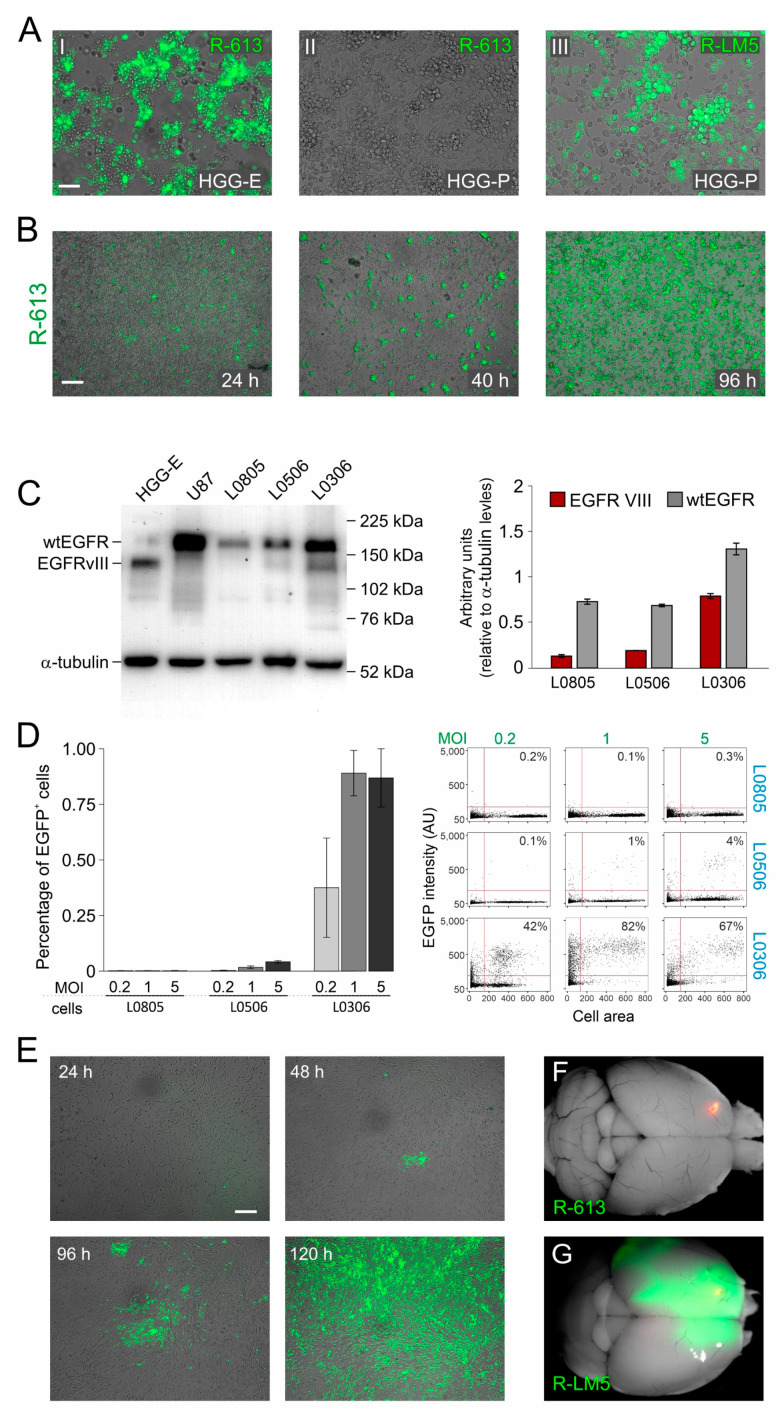
Specificity and safety of oHSV R-613 infection of glioma cells. (**A**) Merged fluorescence and bright-field microphotographs of murine glioma cultures expressing EGFRvIII 96 h after infection by R-613 at MOI 1 PFU/cell (I); expressing PDGF-B 72 h after infection by R-613 at MOI 5 PFU/cell (II); expressing PDGF-B 24 h after infection by R-LM5 at MOI 0.2 PFU/cell (wild-type tropism) (III). (**B**) Merged fluorescence and bright-field microphotographs showing R-613 spread in murine glioma cultures expressing EGFRvIII and infected at MOI 0.3 PFU/cell. (**C**) Quantification of wtEGFR and EGFRvIII protein expression by Western blot in the indicated glioma cell cultures. (**D**) Quantification, based on EGFP expression, of the fraction of human glioma cells infected after 24 h by R-613 at the indicated MOI. Dot blots show a representative experiment. (**E**) Merged fluorescence and bright-field microphotographs showing R-613 spread in EGFRvIII-expressing human glioma cells infected at MOI 0.1 PFU/cell at the indicated time-points. (**F**,**G**) Representative dorsal images of brains from NOD/SCID mice treated with R-613 (**F**) or RLM-5 (**G**) viruses. Scale bars: 25 μm (**A**), 50 μm (**B**,**E**).

Here, we confirmed R-613 specificity for EGFRvIII receptor by using a syngeneic murine glioblastoma model (HGG-E), induced by transplanting INK4a^−/−^ neural progenitor cells overexpressing EGFRvIII in the striatum of BALB/c mice [54]. R-613 was able to infect HGG-E cells in vitro, while it did not infect cells derived from a murine glioblastoma model induced by PDGF-B overexpression (HGG-P). HGG-P and HGG-E were equally susceptible to R-LM5, a wild-type tropism recombinant HSV (Figure 1A). Subsequently, we tested the ability of the oHSV to replicate and perform cell-to-cell spread, an essential feature for oHSV to be effective in therapy. We infected HGG-E cells with R-613 at a low multiplicity of infection and we observed cell-to cell spread throughout the culture within 96 h (Figure 1B).

As a following step, we evaluated the ability of R-613 to infect patient-derived human glioblastoma cells that have endogenous levels of expression of EGFRvIII. We therefore quantified by Western Blot the expression levels of wild type EGFR and EGFRvIII in three cultures of glioblastoma derived from different patients and maintained as glioblastoma initiating cells (hGICs, kindly provided by Dott. Rossella Galli, IRCCS Ospedale San Raffaele, Milan, Italy). HGG-E and U87 human cell line were used, respectively, as positive and negative controls. EGFRvIII was expressed at a high level in L0306 hGICs, at a very low level in L0506 hGICs, and was virtually absent in L0805 hGICs (Figure 1C). Coherently with EGFRvIII expression levels, hGICs were differently susceptible to R-613 infection (Figure 1D). R-613 was able to infect about 90% of L0306 hGICs when inoculated at a MOI of 1 PFU/cell. To exclude the possibility that R-613 entry occurred via the wild-type form of EGFR, typically expressed in neural stem cells (NSCs), we exposed human NSCs to R-613, monitored the cultures up to 96 h and found no infection. In contrast, as expected, NSC were susceptible to the wild-type tropism R-LM5 (Figure S1A). In addition, by monitoring L0306 cells up to 120 h post infection with R-613, we showed that endogenous EGFRvIII level in L0306 cells was sufficient to support cell-to-cell spread (Figure 1E). L0306 hGICs were, therefore, used for all subsequent in vivo experiments.

Before performing in vivo experiments, we decided to evaluate R-613 susceptibility of a wider cohort of patient-derived glioblastoma cells, kindly provided by Dr. Antonio Daga (Ospedale Policlinico San Martino, Genova, Italy). Cells from 14 different glioblastoma patients were infected at 2 different MOI and tested for viral gene expression after 24 h. R-613 was able to infect a significant amount (more than 15%) of total cells in 28% of gliomas at MOI 1 and in 43% of gliomas at MOI 5 (Figure S1B,C). These data gave a hint about the percentage of glioblastoma patients that may potentially benefit of this therapeutic approach.

The safety of R-613 in vivo was evaluated by injecting 2.3 × 10^5^ PFU of purified R-613 virions into the striatum of 5 adult NOD/SCID mice. All mice were killed 16 days after viral injection in the absence of symptoms. Once dissected, their brains showed no evidence of viral encephalitis, nor any trace of the virus other than at the injection site (Figure 1F). In contrast, the injection of 10^5^ PFU of wild-type tropism R-LM5 recombinant virus, performed as a control, induced lethal encephalitis in all the animals within 8 days after injection (*n* = 4, Figure 1G and Figure S1D).

### 3.2. An Early Treatment with R-613 Increases Survival of Human Glioblastoma Bearing Mice by Strongly Delaying Tumor Growth

L0306 cells were engineered to express the secreted Gaussia luciferase (Gluc) and a red fluorescent reporter (DsRed). The reporters expressed by the derived cells (designated L0306-Gluc) allow for the in vivo monitoring of glioblastoma growth by a minimally invasive chemiluminescence assay (as described elsewhere [58]) and an easy identification of tumor cells in autopsy, respectively. In the first experimental setup to test the therapeutic efficacy of R-613, hereinafter referred to as “early treatment”, we infected L0306-Gluc cells in vitro with R-613. After 24 h, we mixed infected cells with non-infected cells to obtain a final population containing 25% of infected cells and 75% of non-infected cells. The mixed cells were injected into the striatum of 18 adult NOD/SCID mice (2 × 10^5^ cells). As a control, we injected the same amount of non-infected L0306-Gluc cells in 26 additional mice. The quantification of Gluc secreted in the bloodstream allowed us to monitor glioblastoma growth during time [58].

**Figure 2:**
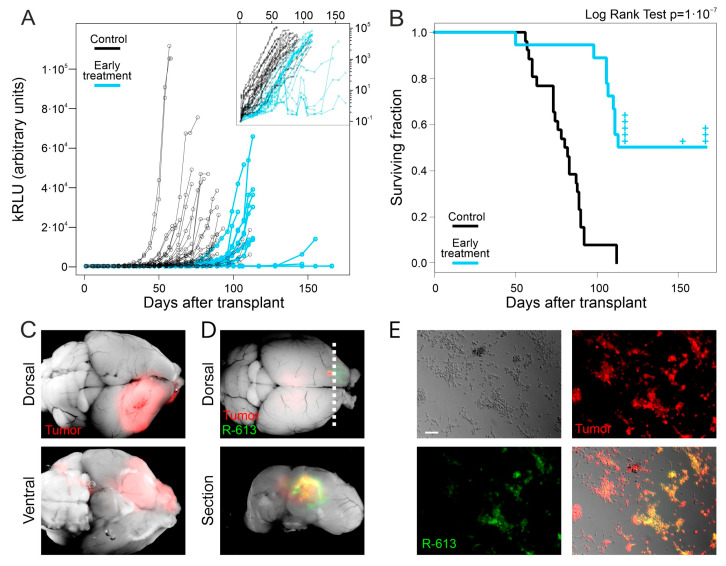
Early treatment with oHSV R-613 on L0306 human glioma cells. (**A**) Levels of Gluc in blood samples over time in mice transplanted with L0306 human glioma cells infected (blue) or not infected (black) with R-613. Each point represents the moving average of the values with a sliding window of 10 days, centered on the indicated time. In the inset, the same curves are in logarithmic scale. (**B**) Kaplan–Meyer survival curves of mice transplanted with L0306 human glioma cells infected (blue line, early treatment) or mock infected (black line, control) with R-613. (**C**) Representative merged fluorescence and brightfield images (dorsal and ventral view) of a mouse brain from the control arm. (**D**) Representative merged fluorescence and brightfield image of a mouse brain from the early treatment arm and corresponding coronal section at the indicated position (dashed line). (**E**) Fluorescence and bright-field microphotographs of murine glioma cells expressing EGFRvIII infected with the filtered supernatant of an in vitro culture of a glioma explanted from the early treatment arm. Scale bars: 25 μm.

Following early treatment, we observed a delayed increase in Gluc levels in all treated animals versus the controls, implying a delayed growth in the tumor mass (Figure 2A). Moreover, treated animals showed a significant increase in the median survival time (114 days after the injection of tumor cells) compared to that of controls (81 days after the injection of tumor cells, Logrank Test: *p* < 10^−7^, Figure 2B). After dissection, mouse brains were analyzed under a fluorescence microscope. As expected, all control animals displayed DsRed positive tumor masses in the injection hemisphere, sometimes marginally relevant infiltrations in the contralateral hemisphere (Figure 2C and Figure S2A). Nine out of the eighteen mice, belonging to the early treatment cohort, died before day 114 and showed overall smaller tumor masses compared to the control group (Figure S2B,C). Notably, we observed EGFP-positive areas, corresponding to R-613-driven gene expression in five tumor masses (Figure S2C). The remaining nine mice treated with oHSV R-613 were analyzed as follows. (i) Some animals (*n* = 5) were sacrificed right after the median time of survival (day 114) to investigate the presence of the tumor or the oHSV in their brains at that time; all of them showed tumor masses and two of them were infected by R-613 as shown by the presence of EGFP-positive areas (Figure 2D); (ii) one mouse died without displaying any symptom nor trace of tumor at day 153, although GLuc levels were slightly increased the days before its death; (iii) three mice were killed at the experimental endpoint (day 167): two of them displayed a very small DsRed positive tumor mass expressing R613-encoded EGFP, coupled to a very slight increase in Gluc level in the blood in the last 10 days of the experimental timeline; one mouse showed only a small DsRed positive area (Figure S2D) and absence of Gluc signal in the blood. Taken together, these data suggest that R-613 is able to infect tumor masses and to restrain glioblastoma development.

We asked whether the failure of R-613 in eradicating glioblastoma could be due to a decrease in R-613 replication overtime. To answer this question, we tested if the EGFP-positive tumors collected after mice death contained infectious R-613 virus. We dissociated the tumor masses from three brains displaying EGFP-positive areas. After one day of culture, the supernatant was filtered through a 0.22 μm filter and added to a culture of HGG-E murine cells expressing EGFRvIII. After 72 h HGG-E cells were EGFP positive, confirming the unharmed replicative ability of R-613 in tumor tissue in vivo even at 113 days post treatment (Figure 2E). Similar results were obtained by adding the filtered supernatant to a culture of human L0306 hGICs in vitro (Figure S2E).

In the EGFP-positive tumors, areas of EGFP-negative cells were evident suggesting the presence of tumor cells still not infected by R-613. Nevertheless, we observed that they could be infected after co-culture with few EGFP-positive cells derived from the same tumor mass (Figure S2F). This result indicates that the presence of R-613 in tumors does not select tumor cells resistant to R-613 infection.

### 3.3. A Late Treatment with R-613 Was Not Effective in Increasing Mouse Survival

As a next step, we tested the efficacy of the EGFRvIII retargeted oHSV R-613 in a more challenging condition, hereinafter referred to as “late treatment”. The late treatment is meant to mimic a therapy whereby the virus would be administered to infect and clear tumor cells left in the resection cavity after surgery. We orthotopically injected the virus 42 days after transplant of 1 × 10^5^ human L0306 glioblastoma cells, a timepoint when tipically the tumor is already established but is still small in volume, based on previously collected Gluc levels quantification data [58]. A cohort of 10 mice received a single treatment, a second cohort of 11 mice received a second treatment 22 days later (64 days after tumor cells transplant). A cohort of control animals (*n* = 5) were transplanted with the tumor cells on the same day and mock treated after 42 days by injecting the culture medium used for virus resuspension (DMEM + 10%FBS).

**Figure 3:**
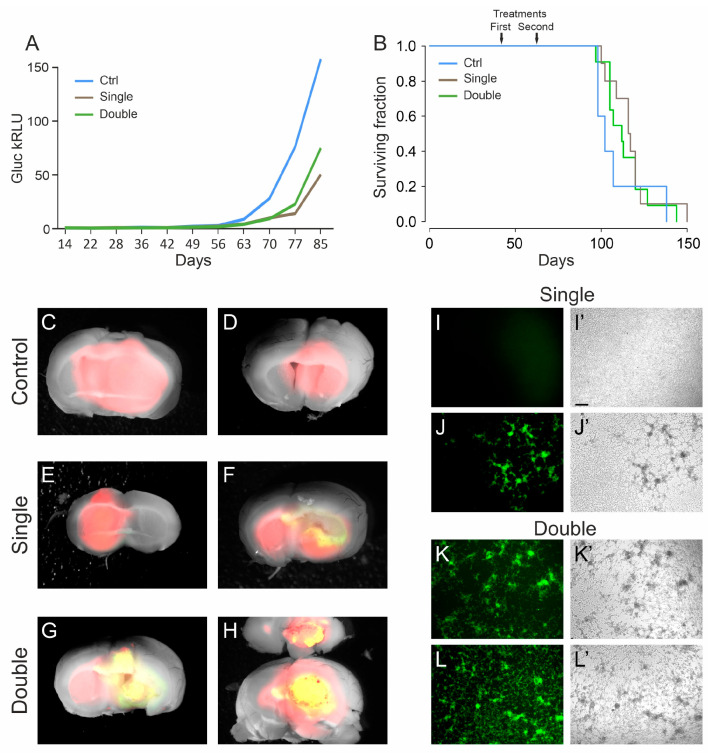
Late treatment with oHSV R-613 on L0306 human glioma cells. (**A**) Median Gluc levels over time for each experimental group measured in blood samples collected starting from 14 days after cell transplant, representing in vivo tumor growth (blue line, control; brown line, single treatment; green line, double treatment). (**B**) Kaplan–Meier survival curves of each experimental arm (same color code as in (**A**)). (**C**–**H**) Merged fluorescence and bright-field micrograph images of two representative brains of mice bearing a DsRed human glioma, for each indicated experimental arm. Yellow areas correspond to DsRed-expressing glioma cells infected by EGFP-expressing R-613. (**I**–**L**) Fluorescence and (**I’**–**L’**) bright-field microphotographs representing murine glioma cultures expressing human EGFRvIII, inoculated with the filtered supernatant from ex vivo cultured human glioma masses. For each indicated experimental arm, two examples are shown. Scale bars: 25 μm.

The analysis of in vivo glioblastoma growth through Gluc quantification showed an initial delay in tumor growth of treated animals compared to controls (Figure 3A). Later, this delay was rapidly recovered and not sufficient to significantly increase the median survival time, neither with single nor with double treatment (117 days and 112 days, respectively) versus that of control animals (102 days; χ-squared: *p* = 0.5) (Figure 3B and Figure S3).

To verify whether tumor masses still contained R-613 infected cells, the brains were dissected and analyzed under a fluorescence microscope. We noticed that almost all the brains from the double treatment arm (8 out of 10) showed EGFP positive cells, while in the single treatment cohort only one out of 10 brains showed EGFP-positive cells (Figure 3C–H). We checked whether the virus was still infective, using the same procedure described above. Four tumors from mice of the double treatment arm were cultured for 24 h and then the filtered supernatants were added to murine glioblastoma cells expressing EGFRvIII. All the supernatants tested were able to efficiently infect the murine cells. We repeated the same experiment with four tumors from mice of the single treatment arm, three EGFP-negative and one EGFP-positive. As expected, only the supernatant derived from the tumor containing visible EGFP-positive cells was able to infect the murine cells (Figure 3I–L). These results confirm that EGFP signal is a bona fide marker of active replication of the recombinant oHSV in the tumor masses, even many days after virus injection (up to 81 days). These data suggest that a double treatment increases the chances to maintain an active oHSV replication in the tumor mass for longer time. Therefore, multiple treatments should be considered for further experiments.

## 4. Discussion

Oncolytic HSVs represent a promising therapeutic strategy against tumors: indeed, immunovirotherapy is based on the ability of viruses to stimulate the immune response of the host against infected tumor cells, and tumors treated with oHSVs become a sort of “agnostic vaccine” against neo-antigens expressed by targeted cells [7,13,14,21]. In a previous work, we showed that R-115, an oHSV fully retargeted to human HER2 and armed with mIL12, is able to specifically infect HER2 positive glioblastoma cells and to induce a potent and long-lasting immune response against tumor cells [39]. Immune response induced by R-115 was able to eradicate HER2 engineered murine glioblastoma in about 30% of orthotopic transplanted immunocompetent mice and to induce the rejection of subsequently transplanted glioblastoma cells, either positive or even negative for HER2 expression. One open question was the efficiency of fully retargeted oHSV in infecting and replicating in patients’ cells, endogenously expressing the target receptor at pathophysiological levels. The present work was purposely designed to fill this gap, and we chose to test an oHSV targeting EGFRvIII endogenously expressed by a relevant fraction of human glioblastomas. Indeed, the oHSV was assayed on orthotopically transplanted human glioblastoma cells. Unfortunately, this experiment does not allow to investigate the protective effects of the immune response, since human cells have to be transplanted in immunodeficient mice. Such an experimental paradigm, lacking the boosting effect of the immune system, is expected to show a blunted immunotherapeutic effect, but it is essential in the perspective of therapy translation to humans. The results obtained in this study show that R-613 is able to efficiently infect and spread in orthotopically xenotransplanted human glioblastoma cells. In the best conditions, R-613 was even able to slow glioblastoma growth alone, without the contribution of immune response.

An important aspect for tumor receptor-retargeted oHSVs is their specificity for tumor cells. Although promising in preclinical studies, results obtained with oHSVs retargeted against HER2 and/or EGFR [15,37] need to be critically evaluated since these receptors are not specific markers for glioma. EGFR, for example, is highly expressed in the healthy brain [59,60], and therefore oHSVs retargeted to EGFR, like KNE, could potentially cause harmful off-tumor effects [15,37]. With this in mind, to increase the specificity of EGFR retargeting, a KNE derivative, KGE-4:T124, was developed. This novel oHSV contains four copies of the miR-124 target sequence at the 3′UTR of the ICP4 gene [44], essential for virus replication. miR-124 is a neurogenic miRNA expressed at high levels in healthy brain, where it should impair KGE-4:T124 replication, and absent in gliomas [42,43],where ICP4 expression can occur and oHSV replication ensue. However, the lack of expression of miR-124 in neural stem cells [45,46] calls for further safety improvements.

An intrinsically safer approach is to target a tumor-specific receptor, or receptor variant, as EGFRvIII, that is not expressed by healthy tissues. To our knowledge, this study reports for the first time on a fully virulent oHSV retargeted exclusively to the highly specific glioblastoma marker EGFRvIII. We demonstrated that R-613 specifically infects EGFRvIII-expressing cells and does not enter neural stem cells expressing the wild-type EGFR receptor. Moreover, R-613 proved to be safe in vivo following injection in murine brain.

It is important to consider that the high specificity of the oHSV for EGFRvIII could limit its use, since glioblastomas are highly heterogeneous, but the EGFRvIII receptor variant is expressed in about one third of these tumors. A relevant proportion of patients could, therefore, be eligible for a therapy, exploiting an EGFRvIII retargeted oHSV [61]. The well-known intra-tumor heterogeneity of glioblastomas is of less concern [62,63] since many studies, as described above, show that the strong immune system activation elicited by oHSV is frequently directed also against tumor neoantigens [7,13,14,21,39]. In this scenario, the possibility of arming R-613 with immunostimulatory cytokines and/or the combinatory use of immune checkpoint inhibitors (ICI) could represent a promising glioblastoma-specific oHSV that overcomes the off-target issues that are typical of previously developed oHSVs.

## Data Availability

Data is contained within the article and supplementary material.

## Supplementary Materials

The following are available online at https://www.mdpi.com/article/10.3390/v13091677/s1, Figure S1: Safety of oHSV R-613; Figure S2: Early treatment with oHSV R-613 on L0306 human glioma cells; Figure S3: Analysis of in vivo glioblastoma growth, Table S1. Features of human and murine glioma cells, Table S2. Features of oncolytic HSVs.
