# Supplementary material for: Specificity, Safety, Efficacy of EGFRvIII-Retargeted Oncolytic HSV for Xenotransplanted Human Glioblastoma
Source: Viruses. 2021 Aug 24;13(9):1677. doi: 10.3390/v13091677 (PMC8473268; doi:10.3390/v13091677)
Supplement: Supplementary file 1 [file viruses-13-01677-s001.zip › viruses-1319865-supplementary.pdf]

viruses

MDPI

Supplementary

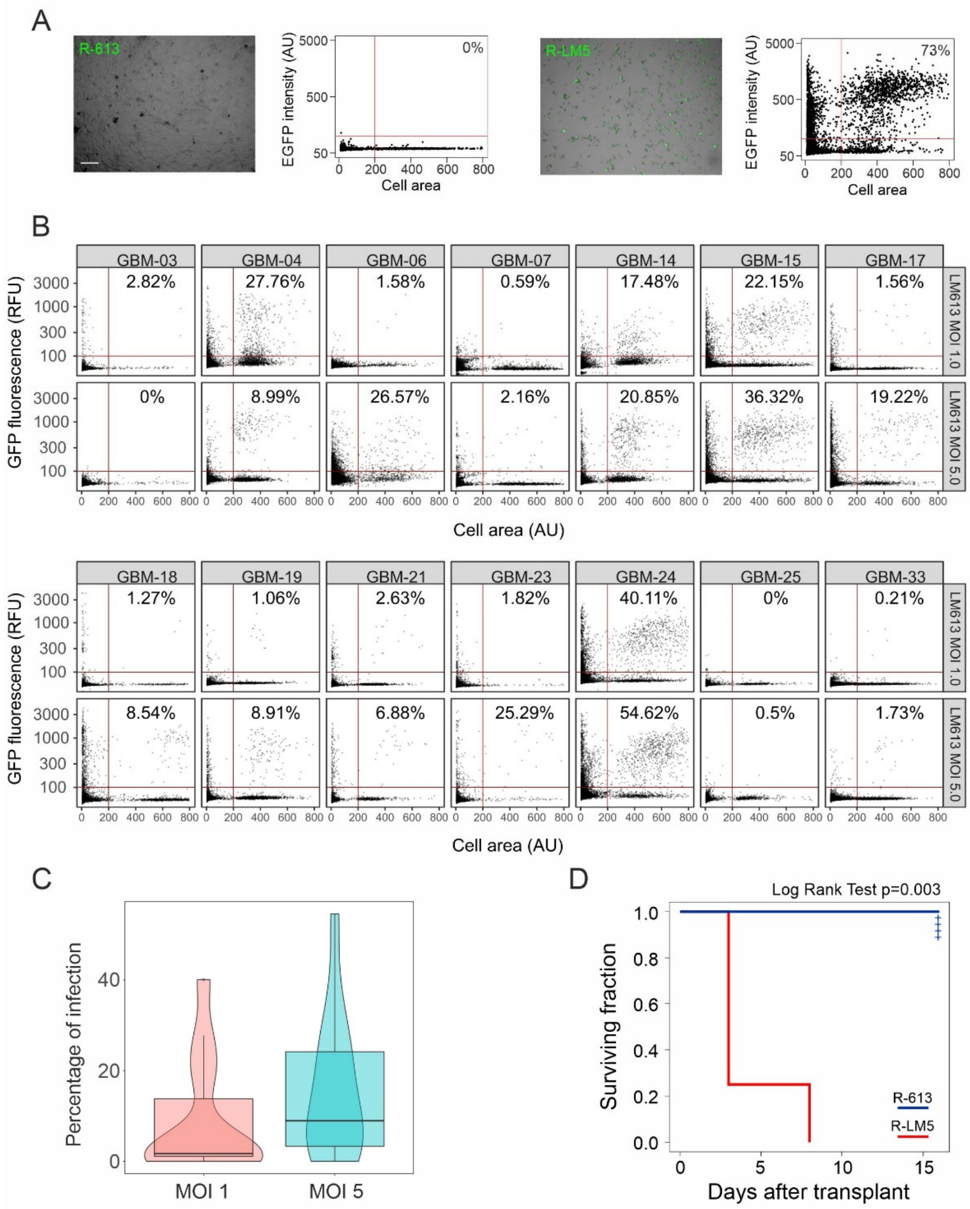

Figure S1. Safety of oHSV R-613. (A) Merged fluorescence and bright-field microphotographs of human NSC cultures infected with R-613 or R-LM5, and corresponding quantifications of the percentage of infected cells based on EGFP expression. (B) Scatterplots showing the quantification, based on EGFP expression, of the percentage of human glioma cells infected after 24 h by R-613 at the indicated MOI. (C) Violin plots showing the distribution of the percentage of human glioma cells infected after 24 h by R-613 at the indicated MOI. (D) Kaplan Meyer survival curves of NOD-SCID mice injected with R-613 (blue line) or R-LM5 (red line). Scale bars: 25 μm.

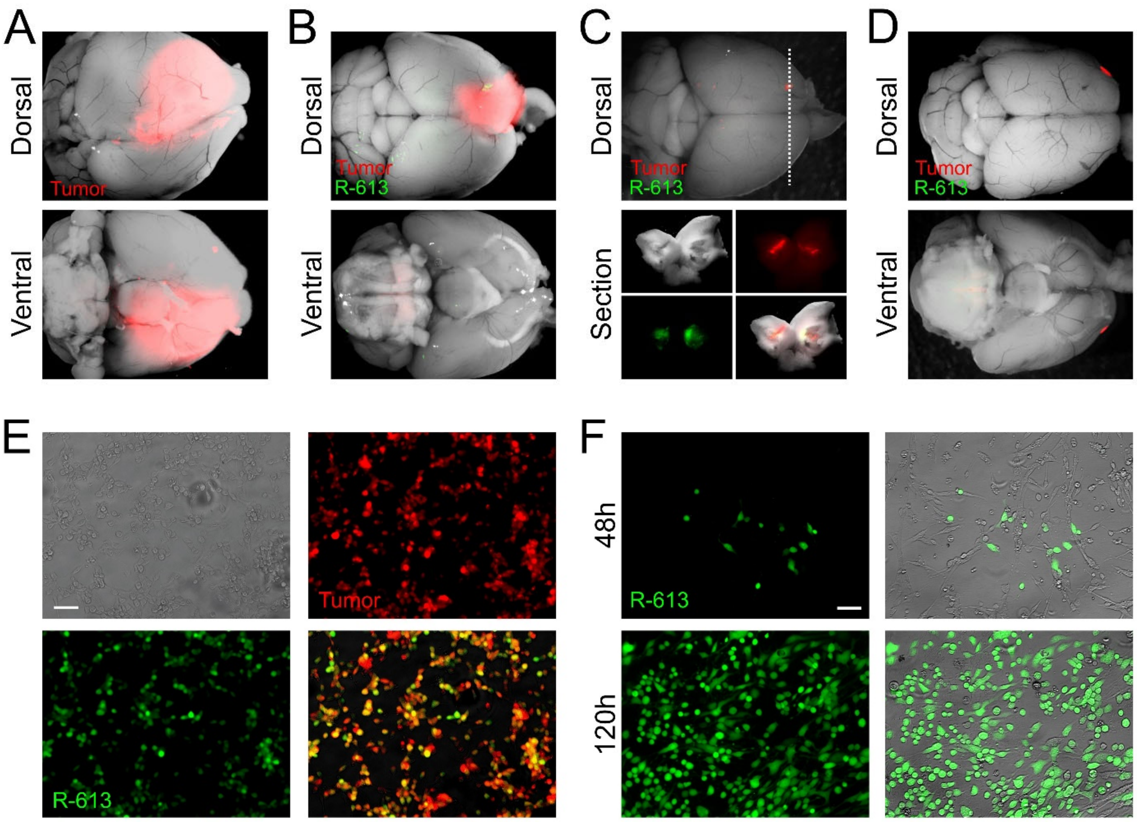

Figure S2. Early treatment with oHSV R-613 on L0306 human glioma cells. (A) Representative merged fluorescence and brightfield images (dorsal and ventral view) of a mouse brain from the control arm. (B-D) Representative merged fluorescence and brightfield images (dorsal and ventral view) of mouse brains from the early treatment arm. Brightfield, fluorescence and merged images of a coronal section at the indicated position (dashed line) is showed in C. (E) Fluorescence and bright-field microphotographs of L0306 human glioma cells infected with the filtered supernatant of an in vitro culture of an explanted glioma from the early treatment arm. (F) Fluorescence and bright-field microphotographs at the two indicated time points of a region of an explanted glioma mass spared *in vivo* by R-613 co-cultured with a small portion of R-613 infected glioma cells derived by the same tumor mass. Scale bars: 25 μm.

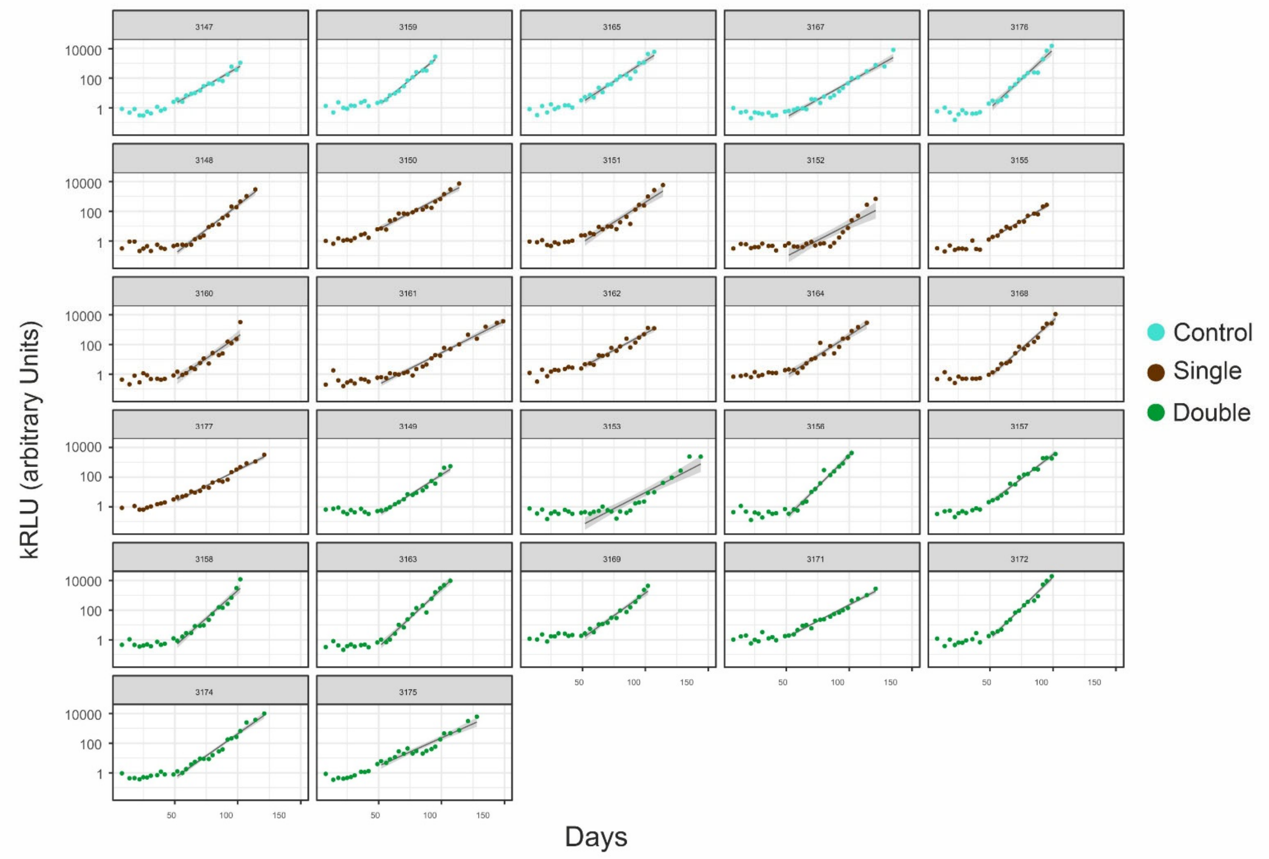

Figure S3. Analysis of in vivo glioblastoma growth. Levels of Gluc in blood samples over time in mice transplanted with L0306 human glioma cells not infected (control, blue lines) or infected with R-613, 42 days (single, brown lines) or 42 and 64 days (double, green lines) after glioma cells transplant.

Table S1. Features of human and murine glioma cells.

Name	Description	Source	Culture Condition	Culture Media	Reference
**L0306**	patient derived glioma cells	Dr. R. Galli IRCCS Ospedale San Raffaele, Milan, Italy	Adherent on matrigel	Neurocult NS-A basal media, Neurocult proliferation supplement, human bFGF, human EGF, heparin	PMID: 20858720, PMID: 15466194
**L0506**	patient derived glioma cells	Dr. R. Galli IRCCS Ospedale San Raffaele, Milan, Italy	Adherent on matrigel	Neurocult NS-A basal media, Neurocult proliferation supplement, human bFGF, human EGF, heparin	PMID: 15466194
**L0805**	patient derived glioma cells	Dr. R. Galli IRCCS Ospedale San Raffaele, Milan, Italy	Adherent on matrigel	Neurocult NS-A basal media, Neurocult proliferation supplement, human bFGF, human EGF, heparin	PMID: 15466194
**U87-MG**	human glioma cell line	commercial	Adherent on plastic	DMEM, 10% FBS	//

**HGG-E**	murine glioma cells induced by transplanting, in the striatum of BALB/c mice, E14 INK4a−/− neural progenitor cells overexpressing EGFRvIII	Dr. P. Malatesta Ospedale Policlinico San Martino, Genova, Italy	Adherent on matrigel	DMEM/F12, B27, human bFGF, human EGF	PMID: 31733287
**HGG-P**	murine glioma cells induced by transducing human PDGF-B into the lateral ventricles of E14 C57Bl/6 mouse embryos	Dr. P. Malatesta Ospedale Policlinico San Martino, Genova, Italy	Adherent on matrigel	DMEM/F12, B27, human bFGF, human EGF	PMID: 19048116, PMID: 19165863
**GBM-03 / GBM-33**	patient derived glioma cells	Dr. A. Daga Ospedale Policlinico San Martino, Genova, Italy	Adherent on matrigel	DMEM/F12, Neurobasal medium, B27, human bFGF, human EGF, heparin	PMID: 19147502

Table S2. Features of oncolytic HSVs.

Recombinant Virus	HSV-BAC Backbone [genetic background]	Reporter [site of insertion]	Modification in gD for Tropism Retargeting	Site of Insertion	Technology for Engineering of gD Locus	References
**R-LM5**	pYEbac102 [HSV-1(F)]	EGFP [in BAC sequences]	none	not applicable	homologous recombination in mammalian cells	PMID: 18684832
**R-613**	pYEbac102 [HSV-1(F)]	EGFP [in BAC sequences]	insertion of scFv to EGFRvIII	gD Δ6–38	galK recombineering	PMID: 29966356
